# Predictors of health-related quality of life (HRQoL) for caregivers of children with developmental disabilities in Saudi Arabia: An observational study

**DOI:** 10.1097/MD.0000000000039206

**Published:** 2024-08-09

**Authors:** Mohammed S. Alghamdi, Abdulaziz Awali

**Affiliations:** a Department of Medical Rehabilitation Sciences, College of Applied Medical Sciences, Umm Al-Qura University, Makkah, Saudi Arabia.

**Keywords:** Caregiver, developmental disabilities, health-related quality of life

## Abstract

To examine predictors of health-related quality of life (HRQoL) for caregivers of children with developmental disabilities, a cross-sectional design was used. Participants were primary caregivers of children with developmental disabilities. Caregivers completed a demographic form about the child and the family, and the Arabic version of Patient-Reported Outcomes Measurement Information System-Profile 29 (PROMIS-29 v2.0). Descriptive statistics were used to report on demographic data, 1-sample *Z* tests to compare PROMIS domain scores with the general population, and multiple linear regression analyses to identify predictors of each domain. Participants were 111 primary caregivers, mostly mothers (65.8%). Caregivers reported higher levels of anxiety, depression, fatigue, sleep disturbance, and pain interference, and lower levels of physical function and social participation compared to the general population, *P* < .05. The regression models for predicting the HRQoL accounted for 12.3% of the variance in the physical function domain (*P* = .016), 13.9% in the anxiety domain (*P* = .009), 24.7% in the ability to engage in social activities and roles (*P* < .001), and 11.4% in the pain interference domain (*P* = .02). In these models, the severity of the child’s disability and/or the child’s age were common significant predictors. Specifically, child’s age was the only significant predictor in 2 domains, the anxiety domain (β = −.29, *P* < .01) and ability to participate in social activities and roles domain (β = .42, *P* < .05). The severity of the child’s disability was the only significant predictor in the physical function domain (β = −.52, *P* < .01). Both the severity of the child’s disability and the child’s age were significant predictors in the pain interference model (β = .40, *P* < .05), and (β = −.23, *P* < .05), respectively. However, the models did not significantly predict depression, fatigue, or sleep disturbance, *P* > .05. HRQoL is a complex construct and is influenced by multiple child and family factors. Implications of the study emphasize the importance of regular HRQoL screening for caregivers, the development of efficient referral systems for support services, and the exploration of respite care options.

## 1. Introduction

Children with developmental disabilities often have complex needs in various life domains, which negatively impact the health and well-being of their caregivers compared to those caring for typically developing children.^[1–6]^ Developmental disability is defined as “a collection of chronic conditions originating in childhood that are manifested as physical, psychological, cognitive, or speech impairments” (pg. 399).^[7]^ The prevalence of developmental disabilities varies based on the type of disability and population being studied. Globally, an analysis of data from the United Nations Children’s Fund (UNICEF) suggests that 10.1% of children under age of 17 years are estimated to have moderate-to-severe disabilities.^[8]^ In the Arab world, the overall estimated prevalence of developmental disabilities among preschool children is 27.5%.^[9]^ In Saudi Arabia, the prevalence of childhood disability is estimated to be 2.7% of the population aged 18 years of age or younger.^[10]^

The transition from institution-based care to home or ambulatory care created demands on family caregivers to care for their child and adapt to continuous childcare needs.^[11]^ Existing literature suggests that caregivers of children with disabilities experience physical problems, mental health challenges, and social challenges.^[1,12,13]^ These challenges contribute to reduced health-related quality of life (HRQoL) for this population.^[14–16]^ Raina and colleague^[11]^ described a model for understanding health and well-being of caregivers of children with disabilities, specifically cerebral palsy. The model emphasizes the importance of considering the broader context, including the characteristics of the child, caregiver and family factors, and the role of social support and coping mechanisms in influencing caregiver health.^[11]^ This model allows for a more holistic understanding of the complex interplay of variables that impact the HRQoL for caregivers of children with disabilities.

In the context of Saudi Arabia, several studies have examined the HRQoL of caregivers for children with various disabilities including cerebral palsy,^[17]^ Down syndrome,^[18]^ autism spectrum disorder,^[19,20]^ and mixed disabilities.^[20–22]^ The collective findings consistently indicate a multifaceted impact on caregivers’ HRQoL, often showing diminished physical, psychological, social, and economic well-being compared to caregivers of typically developing children.^[22]^ While these findings contribute substantially to our understanding of the challenges these caregivers face, there remains a gap in comprehensively understanding the child and family predictors of HRQoL for this population. The aim of the study, therefore, is to examine predictors of HRQoL for caregivers of children with developmental disabilities in Saudi Arabia. This study would provide a better understanding of caregivers’ HRQoL and thereby providing a foundation for developing recommendations for culturally informed interventions to improve HRQoL of caregivers.

## 2. Methods

### 2.1 Study design and participants

A cross-sectional design was used. Participants were primary caregivers of children with developmental disabilities. Inclusion criteria were: primary caregivers (aged over 18 years) of children with developmental disabilities (under the age of 18), and able to read Arabic. Exclusion criteria were: caregivers of children with other diagnoses such as cancer, diabetes, or conditions not classified as developmental disability. Sample size was estimated using G*Power (Version 3.1.9.6). An a priori power analysis was conducted for an *F* test (a fixed model linear multiple regression) with the following parameters: a medium effect size (0.2), an alpha level of (0.05), a power of 0.85, and the number of predictors included (11). The estimated sample size was 104 participants and a convenience sampling technique was employed. The study was approved by the Biomedical Research Ethics Committee at Umm Al-Qura University (Approval No. HAPO-02-K-012-2021-11-850). Participants provided a consent form prior to starting the study.

### 2.2 Tools

Participants completed an online questionnaire that was distributed via social networking platforms. The questionnaire included a demographic form and the Patient-Reported Outcomes Measurement Information System-Profile 29 (PROMIS-29 v2.0).

#### 2.2.1. Demographic form.

A demographic form, designed for the purpose of the study was used to collect data about the child and family. Child-related variables included age, sex, educational level, diagnosis, type of disability, severity of disability. Caregiver variables included caregiver’s relationship to the child, age, social status, education, work status, family income, family household size.

#### 2.2.2. PROMIS-29 v2.0.

The Arabic version of the Patient-Reported Outcomes Measurement Information System- Profile 29 (PROMIS-29 v2.0) was used.^[23,24]^ The PROMIS-29 v2.0 profile measure is designed to assess 7 health-related domains and pain intensity. The 7 domains are as follows: physical function, fatigue, pain interference, depression, anxiety, ability to participate in social roles and activities, and sleep disturbance. Items in each domain are rated on a 5-point Likert scale; however, rating scale is not uniform across items given that these items are from the PROMIS item pool. Raw scores for each domain range from 0 to 20 with higher scores representing lower performance in all domains except for the physical functioning and social participation domains, where higher scores indicate higher performance. The pain intensity item is rated using a single numeric rating item ranging from 0 to 10, with higher scores representing high intensity of pain. The PROMIS-29 v2.0 raw scores for each of the 7 domains are converted to *T* scores using the HealthMeasures Scoring Service powered by Assessment Center^SM^.^[25]^ The validity and reliability of the PROMIS-29 v2.0 has been supported.^[23]^

### 2.3 Statistical analysis

Descriptive statistics were used to report child and caregiver demographics. One-sample *Z* test was conducted to determine if mean *T* score of each PROMIS domain was different from the population mean (50 ± 10). To identify the predictors of each PROMIS domain, multiple linear regression analysis was conducted using demographic variables of the child and caregivers as predictors. In addition, to avoid multicollinearity in the regression analyses, correlations were conducted to determine demographic variables that were significantly associated with each other, and if the correlation exceeded 0.7, one of the variables was excluded from regression analyses. Because the variables severity of disability and family were polytomous (each of them had 3 categories), dummy variables were created and utilized in the regression analysis for 2 categories of those variables. Alpha level was set at 0.05 for all analyses. SPSS (Version 28.00) software was used for all analyses.

## 3. Results

The questionnaire was completed by 111 primary caregivers of children with disabilities. The demographics of those participants are summarized in Table 1. The majority of the caregivers were mothers (65.8%), married (80.2%), had undergraduate degree or higher (56.8%), did not work (67.7%), and their mean age was around 40 years old. For children, the majority of them were at preschool or school levels (71.2%) with a mean age of children was 9 years, and the majority had non-motor disability (58.6%) at moderate severity level (58.6%). Table 1 describes the demographic information of the children with disability.

**Table 1:** Demographic information of caregivers and children.

Variables	Description	Data (n = 111)
Caregiver relationship to child	Mother	73 (65.8%)
	Someone other than the mother	38 (34.2%)
Caregiver age	Mean (SD)	40.1 (10.0)
Social status	Married	89 (80.2%)
	Not married	22 (19.8%)
Caregiver education	High school or less	48 (43.2%)
	Undergraduate or further	63 (56.8%)
Caregiver work status	Does not work	75 (67.6%)
	Work (part-time or full-time)	36 (32.4%)
Family income	<6000	31 (27.9%)
	6000 to 12,000	58 (52.3%)
	More than 12,000	22 (19.8%)
Family household size	Mode (min-max)	5 (2–12)
Child age	Mean (SD)	9. 26 (5.0)
Child sex	Male	67 (60.4%)
	Female	44 (39.6%)
Type of disability	Motor	46 (41.1%)
	Non-motor disability	65 (58.6%)
Associated disability status	Yes	54 (48.6%)
	No	57 (51.4%)
Level of disability	Mild	19 (17.1%)
	Moderate	65 (58.6%)
	Sever	27 (24.3%)
Child education level	No school	32 (28.8%)
	Preschool or school	79 (71.2%)

Regarding the PROMIS *T* score, participants rated their anxiety, depression, fatigue, sleep disturbance, and pain interference higher (worse) than the mean of the general population. In addition, participants rated their physical function and their ability to participate in social roles and activities lower (worse) than the mean of the general population. Table 2 provides a comparison between sample PROMIS *T* score and population mean.

**Table 2:** Comparison between sample PROMIS T-score and population mean.

Domain	Mean T-score (SD)	*Z* statistic	*P* value
Physical function	44.2 (9.2)	−6.11	*P* < .01
Anxiety	61.5 (8.6)	12.16	*P* < .01
Depression	58.6 (8.9)	9.09	*P* < .01
Fatigue	59.1 (8.8)	9.55	*P* < .01
Sleep disturbance	53.0 (6.6)	3.14	*P* < .01
Ability to participate in social roles and activities	44.0 (8.5)	−6.30	*P* < .01
Pain interference	60.3 (8.7)	10.85	*P* < .01
Pain Intensity	3.0 (1.3)	–	–

Table 3 presents the regression models for each domain. The regression analysis for predicting the physical function domain indicated that the model accounted for 12.3% of its variance (*F*(13, 110) = 2.18, *P* = .016), with the severity of disability being a significant contributor. The model explained 13.9% of the variance in the anxiety domain (*F*(13, 110) = 2.36, *P* = .009), with child age as the only significant variable. In regards to predicting the caregivers’ ability to engage in social activities and roles, the model accounted for 24.7% of the variance (*F*(13,110) = 3.78, *P* < .001), with child age as the only significant variable. For the prediction of the pain interference domain, the model explained 11.4% of the variance (*F*(13,110) = 2.09, *P* = .02), with child age and severity of disability being significant predictors. However, the model did not significantly predict depression (*F*(13, 110) = 1.62, *P* = .093), fatigue (*F*(13,110) = 1.62, *P* = .098), or sleep disturbance (*F*(13, 110) = 1.60, *P* = .098).

**Table 3:** Predictors of caregivers’ quality of life using PROMIS *T* score

	Physical function	Anxiety	Depression	Fatigue	Sleep Disturbance	Ability to participate in social activities and roles	Pain interference
Child age	β = .10	**β =** −.29**	**β =** −.25*	**β =** −.33**	β = −.10	β = .42*	**β =** −.23*
Child sex	β = −.09	β = −.02	β = −.02	β = .02	β = .13	β = −.06	β = .02
Type of disability	β = .02	β = .20	β = .21	β = .07	β = .18	β = −.10	β = .10
Additional disability	β = −.14	β = .13	β = .08	β = .02	β = .13	β = −.19	β = .10
Child education level	β = −.17	β = .12	β = .03	**β** = −.23*	β = −.13	β = .12	β = .01
Caregiver relation to child	β = .13	β = −.07	β = −.00	β = .01	β = .18	β = .10	β = −.04
Caregiver’s social status	β = .01	β = −.15	β = −.10	β = .08	β = .12	β = −.04	β = .03
Caregiver educational level	β = .02	β = .17	β = .18	β = .05	β = .08	β = .01	β = −.19
Caregiver work	β = −.14	β = −.04	β = −.10	β = −.06	β = .06	β = .02	β = .19
Family income (between 6 thousands to 12 thousands Saudi Riyals/mo)	β = .19	β = −.05	β = −.12	β = −.02	**β =** −.26*	β = .04	β = −.19
Family income (above 12 thousands Saudi Riyals/mo)	β = .17	β = −.13	β= −.12	β = .05	β = −.31*	β = −.11	β = −.17
Severity of disability (Moderate)	**β** = −.31*	β = .12	β = .14	β = .19	β = .17	β = −.07	β = .30*
Severity of disability (Severe)	**β** = −.52**	β = .21	β = .24	β = .16	β = .11	β = −.25	β = .40**
**Model significance**	.016**	.009**	**.093**	**.093**	**.098**	.001**	.021**
**Adjusted *R*^2^**	**.123**	**.139**	**.068**	**.178**	**.066**	**.247**	**.114**

Bold values indicates significant values with *P < .05 and **P < .01.

## 4. Discussion

The aim of this study was to examine predictors of HRQoL for caregivers of children with developmental disabilities in Saudi Arabia. Compared to the general population mean, caregivers in this study reported high scores in the anxiety, depression, pain interference, sleep disturbance and fatigue domains and lower scores in physical function and social participation domains. Consistent with our findings, several studies found reduced HRQoL for Saudi caregivers of children with autism spectrum disorder (ASDs),^[19,20,26]^ cerebral palsy (CP),^[17]^ Down Syndrome (DS),^[18]^ and mixed disabilities.^[27]^ In contrast, Alwhaibi et al^[28]^ observed no significant difference in overall HRQoL between mothers of children with and without CP/DS. These findings highlight the impact of caregiving for a child with disability on caregivers’ mental and physical well-being.

Our finding that the severity of the child’s disability is a significant contributor to the physical function of the caregiver might be attributed to the demands of caregiving. The model’s ability to account for 12.3% of the variance in the physical function domain, while modest, highlights the complexity of HRQoL. The unique challenges of caregiving for a child with severe disability, such assisting the child with self-care activities, managing medical equipment, or frequent healthcare visits, can contribute to physical exhaustion and strain of caregivers. Current studies on HRQoL for caregivers of children with disabilities in Saudi Arabia show that caregivers have reduced HRQoL in the physical domain.^[17,18,20]^ However, none of these studies have studied the role severity of disability in predicting HRQoL. In contrast, evidence shows that severity of child’s disability, especially in children with CP, is associated with high caregiver’s burden and reduced HRQoL.^[29,30]^

Research has consistently shown a high prevalence of depression and anxiety symptoms among parents of children with intellectual and developmental disabilities.^[31]^ In Saudi Arabia, for instance, around 85% of caregivers of children with neurodevelopmental disorders were found to experience anxiety and depression.^[32]^ Our study aligns with these findings, as the regression model explained 13.9% of the variance in anxiety reported by caregivers, with the child’s age being a significant predictor. Interestingly, our model did not find a significant correlation with depression, which contrasts with the broader trend in the literature. The increasing demands for children at a younger age may contribute to caregivers’ anxiety. This is supported by studies indicating that factors such as the severity of the child’s disability and lower household income are associated with higher levels of depressive symptoms among parents.^[31]^ However, contrary to our findings, previous studies found other factors associated with depression and anxiety in caregivers including number of children with disabilities, presence of chronic conditions and the caregiver’s educational level.^[32–34]^

Participation in social activities is an important outcome for children with disabilities.^[35]^ However, the involvement and experiences of caregivers in these activities often remain under-studied in the literature. Existing research indicates that in Saudi Arabia, caregivers of children with disabilities often report low ratings in the social functioning aspect of HRQoL.^[22,28,36]^ In our study, child’s age accounted for 24.7% of the variance in caregiver’s ability to participate in social activities and roles. This may indicate that as children grow older, the demands on caregivers typically change, possibly becoming less, thus allowing more time and flexibility for caregivers to engage in social activities. However, since this factor accounts for modest variance, other factors may also play a role in determining a caregiver’s social participation. Previous studies reported factors such as presence of support systems and coping strategies of caregivers, and family functioning.^[37,38]^

Caregivers of children with disabilities often experience pain that is attributed to caregiving burden.^[20,39,40]^ In our study, we found that a child’s age and the severity of their disability significantly influence the degree to which caregivers experience pain interference, explaining about 11% of the variance observed. This implies thata younger age of children and a higher severity of disability are associated with heightened challenges for caregivers in performing household chores, managing day-to-day activities, accomplishing work around the home, and participating in social activities. Our findings are consistent with those of Neves et al^[41]^ in which they reported that caregivers of children with CP who had minor motor impairments generally report fewer average symptoms of low back pain. In addition, Castillo et al^[39]^ found that caregivers who are obese, suffer from a disability, or have a mental health condition are more likely to experience chronic non-cancer pain.

In contrast to the existing literature, our study found no significant predictors for fatigue or sleep disturbance among caregivers. The lack of significant findings in our study might be attributed to the nature of factors included, which was limited to basic characteristics of the child and caregivers. It is possible that the presence of caregiver disrupted sleep routines, often a direct result of attending to the child’s needs during the night. This is particularly evident in caregivers of children who depend on medical technology, where sleep disruption is common due to the high level of care needed.^[42]^ The burden and mental health of the caregiver, such as depression and anxiety, have been identified as significant predictors of their sleep quality and fatigue levels.^[43]^ Additionally, the physical health of caregivers can impact their sleep, with factors like chronic fatigue syndrome being prevalent among caregivers of children with cerebral palsy.^[44]^ Furthermore, the sleep problems of the child, such as frequent awakenings or restlessness, are directly linked to the caregiver’s sleep patterns, leading to increased fatigue.^[45]^ These findings suggest that addressing sleep disturbances and fatigue in caregivers of children with disabilities necessitates a comprehensive approach that considers a range of psychological, physical, and environmental factors.

The collective findings of our study offer implications for improving HRQoL of caregivers of children with disabilities in Saudi Arabia. First, we recommend the regular screening of caregivers’ HRQoL during their children’s medical appointments and visits to rehabilitation centers. This proactive approach can facilitate early detection and intervention for stress or health issues in caregivers. Second, an efficient referral system is essential to address the physical and psychological challenges caregivers may face. This would ensure that caregivers receive timely and appropriate support from healthcare or mental health services. Last, we propose exploring the implementation of respite care in Saudi Arabia. Evidence shows that respite care offers significant benefits, providing caregivers with necessary breaks and reducing the risk of burnout.^[46,47]^

There are some limitations in this study. First, the predictors used in this study included basic characteristics of the child and caregiver, which might not have provided a full understanding of the HRQoL of this population. Second, we used a convenience sampling method. While this approach facilitated data collection, it may limit the generalizability of the findings due to potential biases inherent in nonrandom sampling methods. Last, the study included caregivers of children with different disabilities, and we were not able to conduct analyses for each group due to the limited number of participants. Future research should aim to investigate a broader range of determinants of HRQoL, encompassing child, family, caregiver, and environmental factors. Additionally, future studies should focus on developing and testing interventions and implementation strategies that can effectively support caregivers and improve their HRQoL. Such studies will help to translate the existing knowledge into practical solutions and provide evidence-based recommendations for improving the well-being of caregivers. Furthermore, studies with a larger sample size would enhance the ability to conduct subgroup analyses and provide a more comprehensive understanding of the HRQoL across different types of disabilities.

## 5. Conclusion

This study highlights the multifaceted impact of caregiving for children with developmental disabilities on the HRQoL of caregivers in Saudi Arabia. Caregivers in this study reported high scores in the anxiety, depression, pain interference, sleep disturbance and fatigue domains and lower scores in physical function and social participation domains. The severity of the child’s disability and the child’s age emerged as significant predictors in the majority of HRQoL domains. The collective findings of our study offer implications such as regular screening of caregivers’ HRQoL during children’s medical appointments, efficient referral systems for support services, and the exploration of respite care options to alleviate the challenges faced by these caregivers. Future research should aim to investigate a broader range of determinants of HRQoL, encompassing child, family, caregiver, and environmental factors. Additionally, future studies should focus on developing and testing intervention and implementation strategies that can effectively support caregivers and improve their HRQoL.

## Author contributions

**Conceptualization:** Mohammed S. Alghamdi.

**Investigation:** Mohammed S. Alghamdi, Abdulaziz Awali.

**Methodology:** Mohammed S. Alghamdi, Abdulaziz Awali.

**Resources:** Mohammed S. Alghamdi, Abdulaziz Awali.

**Software:** Mohammed S. Alghamdi, Abdulaziz Awali.

**Supervision:** Mohammed S. Alghamdi.

**Validation:** Mohammed S. Alghamdi, Abdulaziz Awali.

**Writing – original draft:** Mohammed S. Alghamdi, Abdulaziz Awali.

**Writing – review & editing:** Mohammed S. Alghamdi, Abdulaziz Awali.

**Data curation:** Mohammed S. Alghamdi, Abdulaziz Awali.

**Formal analysis:** Abdulaziz Awali.

**Project administration:** Abdulaziz Awali.
